# Updates on the Role of Spinal Cord Stimulation in the Management of Non-Surgical Chronic Lower Back Pain

**DOI:** 10.7759/cureus.18928

**Published:** 2021-10-20

**Authors:** Vinayak Aryal, Sujan Poudel, Fizza Zulfiqar, Thakur Shrestha, Annie Singh, Shahtaj A Shah, Umar Soomro, Jinal Choudhari, Jonathan Quinonez, Samir Ruxmohan, Amean Amra, Trevine Albert, Jesse Kemmerlin, Joel Stein

**Affiliations:** 1 Division of Research & Academic Affairs, Larkin Community Hospital, South Miami, USA; 2 Pathology and Laboratory Medicine, Nepal Cancer Hospital and Research Center, Lalitpur, NPL; 3 Department of Neurology/Osteopathic Neuromuscular Medicine, Larkin Community Hospital, South Miami, USA; 4 Department of Neurology, Larkin Community Hospital, South Miami, USA; 5 Osteopathic Neuromuscular Medicine/Family Medicine, Larkin Community Hospital Palm Springs Campus, Hialeah, USA; 6 Division of Interventional Pain, Larkin Community Hospital, South Miami, USA; 7 Division of Pain Management, Larkin Community Hospital, South Miami, USA

**Keywords:** stimulation, spinal cord, low back pain, chronic, spinal cord stimulation

## Abstract

Studies have shown that spinal cord stimulation (SCS) therapy is effective in the management of chronic low back pain. It plays a role by minimizing the intensity of chronic pain, improving the quality of life index, reducing the intake of narcotic analgesics, and increasing the functional improvement in the working environment. However, spinal cord stimulation therapy is not universal because of the complications in the procedure itself, the invasive nature of the treatment, and cost-effectiveness. Therefore, the proper selection of the patients is necessary to get the maximum benefit from the treatment. The study's main objective is to determine the role of spinal cord stimulation in treating non-surgical patients with chronic low back pain. The article will review the mechanism, outcomes, efficacy, predisposing factors in the success and failure of the treatment and indications, contraindications, and selection of patients undergoing spinal cord stimulation therapy. A manual search of the literature was done using databases like Google Scholar and PubMed using the keywords: spinal cord, stimulation, chronic, and low back pain. A total of 37 articles were included in the study after considering the inclusion and exclusion criteria. Spinal cord stimulation therapy effectively treats refractory lower back pain, considering the technology and mechanism of action. The authors conclude that spinal cord stimulation therapy can be used to manage chronic low back pain, other neuropathic pain, and ischemic pain when other standard treatment methods have failed and the pain persisted for more than six months.

## Introduction and background

Chronic lower back pain is a common comorbidity worldwide and the fifth most common in the United States [1]. In the majority of those who struggle with low back pain, the cause cannot be identified, thereby classifying the pain as non-specific. As this condition is common worldwide, the current treatment recommendations vary between countries. In respect to the large population of people with chronic, non-specific, lower back pain ineligible for surgery, there is a need for effective treatment options. Spinal cord stimulation (SCS) therapy is a commonly practiced procedure in medicine to treat chronic lower back pain [2]. Spinal cord stimulation is a well-accepted form of treatment of neuropathic pain. SCS therapy is a modern form of pain management treatment to reduce chronic back pain with different types of causation. SCS does not change the body's anatomy and the pain pathway, unlike the surgical method of managing the pain [3].

The gate theory of pain proposed by Melzac and Wall in 1965 stated that the large myelinated nerve fibers in the dorsal column inhibit the transmission of impulses to the smaller and unmyelinated afferent nerves in the spinal cord [4]. Experimentally the gate theory was tested using surgically implanted electrodes to stimulate the dorsal columns in treating back pain by Shealy and colleagues in 1967 [5]. After the experiment, it was clear that the implanted electrodes stimulate the dorsal horn and roots of the spinal cord in ablating the neuropathic back pain. Recent studies and clinical research suggest that the impulses of pain transmission through the spinothalamic tract and central mechanism are inhibited by the SCS therapy by a direct influence on the afferent neurons by releasing various neurotransmitters [6].

SCS has been used widely for chronic back pain over pharmacological or surgical treatments, as it has shown favorable outcomes. Many studies and trials have shown the promising results of SCS in improving chronic lower back pain [4]. Efficient treatment modalities are a large clinical need in the present time to treat non-surgical chronic lower back pain. The study's main objective is to determine the role of spinal cord stimulation in treating non-surgical patients with chronic low back pain. The article will give a basic review of the mechanism, outcomes, efficacy, predisposing factors in the success or failure of the treatment, and indications, contraindications, and selection of patients undergoing spinal cord stimulation therapy.

A systematic search was carried out of the literature and relevant articles in databases like Google Scholar, PubMed Central, and Web of Science. The keywords used to search the articles were spinal cord, stimulation, and low back pain. We prepared a separate Microsoft Excel sheet (Microsoft Corporation, Redmond, WA) of the searched literature and did the initial screening by reviewing our inclusion and exclusion criteria. The included patients were more than 18 years old, with symptoms of lower back pain of more than six months and without a history of surgical treatment. In addition, patients with previous surgical treatment, patients with psychiatric illness who are not in a state of evaluating the treatment outcome, and patients with mechanical instability were excluded from the study. The articles that met the exclusion article during the screening and the duplicates were removed. A total of 37 articles were included, and we reached a consensus with a discussion with a third independent author.

## Review

Mechanism of pain relief using SCS

Spinal cord stimulation (SCS), previously also known as dorsal column stimulation, is mechanistically based on "gate control theory for pain transmission" [7]. In 1965, a scientific theory was published by Melzack and Wall, describing the dorsal horn as a portal of entry for pain signals and the role of central neural pathways regulating pain signals. The theory explains that the gate opens when there is an activation of thin fibers (A-δ and C) by a noxious stimulus that causes the perception of pain, whereas, large-diameter somatosensory afferent fibers (A-β) form collaterals in the dorsal horn of the spinal cord, leading to inhibition of the transmission of pain signals when activated [8]. The electrical stimulation of the spinal cord can be divided into three groups depending on the anatomical location targeted for pain relief (a) peripheral, i.e., distal to the dorsal root ganglion; (b) spinal cord, dorsal root ganglion; and (c) supraspinal structures [9]. The latest available techniques for SCS involves percutaneous insertion of electrodes into the epidural space via a modified Tuohy needle under imaging guidance, and the analgesic effect is mediated by gamma-aminobutyric acid (GABA), although there is also a rise of other neurotransmitters in the dorsal horns of the spinal cord, including serotonin, substance P, glycine, and adenosine [1,7]. The conventional SCS involves introducing percutaneous leads into the epidural space while undergoing laminectomy or laminotomy procedures; paddle leads with four to 16 electrodes are inserted [10]. Percutaneously SCS leads are mostly are placed in the epidural space (midline) to stimulate the dorsal column tracts of the spinal cord.

Application of electrical stimulation activates the dorsal column, which leads to pain inhibition, the primary mechanism of conventional (tonic) SCS [10-11]. It leads to depolarization of primary afferent fibers, eliciting presynaptic inhibition in dorsal horns. The effect observed results in abrupt cessation of discharges and inhibition of nociceptive signals in the deep lamina wide dynamic range (WDR) neurons of dorsal horns of the spinal cord [9]. The dorsal root ganglion (DRG) neurons are pseudo unipolar and located in the lateral epidural space bilaterally in the spinal cord, at the distal end of the dorsal within fixed bony vertebral structures (neuroforamen) [11-12]. The DRG contains the cell bodies of sensory neurons and has an effective role in controlling pain. The implantation of leads for DRG stimulation involves placing the lead into the neural foramen close to DRG [11]. After epidural access, a unique delivery system comprising a curved sheath is advanced through the en-bloc needle technique toward the contralateral pedicle, and another lead is advanced through the foramen, ensuring electrodes are positioned near the DRG [12]. Nashold et al. were the first to describe supraspinal involvement in pain transmission and pain relief by the stimulation of dorsal fasciculi of the spinal cord. It further suggested that because of the cerebral level processing of pain signals involving the diencephalon and brainstem, the spinal cord stimulation "masks" neuropathic pain but not nociceptive pain [13]. Numerous studies in the previous years have reported effective supra spinal stimulation by somatosensory evoked potentials pain relief [14]. Stiller et al. explained the mechanisms of spinal cord stimulation utilizing microdialysis catheter techniques showing markedly reduced levels of γ-aminobutyric acid (GABA) in freely moving rats after the spinal cord stimulation, particularly involving the peri-aqueductal gray area region [10,15].

Thus, the mechanism of SCS therapy depends on the gate control theory where the noxious stimulus stimulates the smaller fibers to cause the pain, and the large diameter somatosensory fibers lead to the inhibition of the transmission of the pain signals forming collaterals in the dorsal horn after activation. Different anatomical locations like distal to the dorsal root ganglion, dorsal root ganglion, and supraspinal structures are used to place the electrodes for electrical stimulation of the spinal cord.

Outcome of spinal cord stimulation therapy

Chronic lower back pain is a disorder that affects all aspects of the individuals' life afflicted by it. In treating lower back pain in non-surgical patients, spinal cord stimulation has been a well-established treatment option [16]. Our study discusses the primary and secondary outcomes in patients after treatment with SCS and compares the patient status at baseline and certain months after the intervention. The main primary outcome to be considered is reducing the pain intensity by using the visual analog scale (VAS) [17]. The general characters of the secondary outcome are the Oswestry Disability Index (ODI score), European quality of life index (EuroQol five), Dimensional Questionnaire (EQ-5D), and the global impression of change, satisfaction, reduction in pain medication [18]. In the study done by Chapman et al., the overall decrease in patients' pain scores is 80% in one month of intervention. There is a mean 77.8% reduction relative to baseline, with around 50% pain relief in all patients. Out of 17 patients, nine reported 80% or more pain relief [1]. In the study done by Al Kaisy et al., there was a significant decrease in VAS score (1-10) from a mean of 79 ± 2 mm to 10 ± 12 mm. More than 60 % reported greater than 50% pain reduction [19]. Likewise, another study done by Carolis et al. showed a decrease in VAS score (1-10) from the mean of 7.8 ± 1.3 to 2.5 ± 2.1 after 19.9 months; the average pain relief percent was 70 ± 24 % [20]. The reduction of pain intensity in the study carried out by Stidd et al. in seven patients, 89% reported > 50 reductions in lower back pain [21]. In the same way, in the study of primary outcomes in 21 patients in the study done by Vallejo et al., there was a decrease of mean VAS score from 79 ± 12 mm to 10 ± 12 mm at 36 months. There was a greater than 50% reduction in pain intensity. The response rate was 58% from baseline in three months [22].

In the study by Chapman et al. in 17 patients, the ODI score improved from 67.7 ± 14.2 at baseline to a mean of 18.8 ± 11.5 after one month of treatment and 10.0% ± 8.4 after six months of treatment. The EQ-5D index increased from a mean of 0.30 ± 0.16 to a mean of 0.85 ± 0.10 after one month and 0.86 ± 0.07 after six months of treatment [1]. In the study by Chapman et al., the ODI score was reduced from 67.7% ± 14.2 to 14.7 ± 13.1 at 12 months. Among them, 10 did not need a spinal injection after treatment, three decreased the use of opioid analgesics, and two completely left using opioid analgesics. In the study by Alkaisy et al., the ODI score improved from 53 ± 13 at baseline to a mean of 19.8±13 after 12 months. Similarly, the EQ-5D improved from 0.17 to 0.84 after 36 months of treatment by SCS. More than 85% of patients were satisfied with the treatment [17,22]. Improvement in the EQ-5D from baseline to absolute 0.213 ± 0.025 (88%), ODI score from baseline to absolute is 13 ± 42.2 improvements, and the percentage of patient satisfaction was 74% after 90 days in the study carried by Vallejo et al. [22].

Thus, the primary and secondary outcomes in the patients after SCS therapy in the different studies show that it is effective in treating patients with lower back pain in non-surgical patients. The primary and secondary outcomes of the spinal cord stimulation therapy in different clinical trials are shown in Table 1.

**Table 1:** The primary and secondary outcomes of the spinal cord stimulation therapy in different clinical trials VAS: visual analog scale, NPRS: numerical pain rating scale, NRS: numerical rating scale, ODI: Oswestry disability index, HF 10 therapy: High-frequency spinal cord stimulation at 10 kHz, MME: morphine milligram equivalents

No.	Last name of the first author/year	Country	Type of Intervention	No. of patients enrolled	Mean age of the patients (Mean±SD)/Years	Follow-up/Months	Pain reduction intensity		ODI scores of disability		Medication intake		Patient satisfaction/Response	European quality of life index		Adverse effects
							Baseline	After invention	Baseline	After intervention	Baseline	After intervention				
1.	Al-Kaisy et al. / 2018 [17]	Finland	10-kHz high-frequency SCS	21	43.1±9.6	36	VAS score: 79±12mm	10±12 mm (80%) reported more than 50% pain reduction	53±13	19.8±13	10% not used in the baseline	88% of all subjects were not using	85% satisfied	0.17	0.84	1-mild pain in the IPG site. No other adverse effects.
2.	Benyamin et al. / 2020 [23]	North America	1,000 Hz frequency with 90 μs pulse width followed by 300 Hz frequency with 800 μs pulse width	64	57.5 ± 12.9	3	NRS-11 for pain-7.5	NRS-11-3.8	51.5	32.1	N/A	N/A	84% satisfied	0.58	0.74	40% of the patients experienced adverse effects.
3.	Russo et al. / 2016 [24]	Australia	HF10 SCS	256	55 ± 16	7.5± 1.5	NPRS:7.5 ± 1.6	3.0 ± 1.5; NPRS was reduced by 60% (P ≤ 0.001)	41.4	32.8(8.7 point reduction at 6 months)	N/A	N/A	72%	N/A	N/A	No severe adverse effects.
4.	Al-kaisy et al. / 2020 [25]	Europe and USA	HF10 SCS	89 SNEZA RCT and SNEZA EU: 67	N/A	12	SNEZA RCT: back pain: 7.2 (0.3) cm SENZA EU back pain 8.1 (0.2) combined: back pain 7.7 (0.2)	SNEZA RCT group: 75% responders for back and leg pain, SNEZA EU group: 71% responders for back and leg pain Combined: 73% responders months for back and leg pain an average of 5.6 cm (p < 0.0001).	Combined ODI score:52.3	After 13 months ODI Score:36.6, (decrease in ODI scores of 15.7% points from baseline)	85.3 MME	Decreased to 39.8 (12.9) MME	73%-responder rate.	N/A	N/A	No severe adverse effects.
5.	Carolis et al. / 2017 [20]	USA	HF10 SCS	61	56 ± 12	19.4 ± 8.6	7.9 ± 1.3	2.5 ± 2.1	N/A	N/A	N/A	N/A	N/A	N/A	N/A	N/A
6.	Al-Kaisy et al. / 2014 [19]	UK, Belgium	HF10 SCS	65	50.8 ± 9.2	24	8.4 ± 0.1.	3.3 ± 0.3, 60% of the implanted patients had at least 50% back pain relief	55 ± 1	40 ± 2	86% using opioid analgesics	Reduced to 57% after 24 months	81%	N/A	N/A	Pocket pain and lead migration
7.	Chapman et al. / 2019 [1]	USA	HF10 SCS	17	57	12	Pain score: 92.5 ± 8.0 mm	Pain score: 19.3 ± 16.4 mm with 77.8% pain reduction.	67.7% ± 14.2	14.7 ± 13.1	13(100%) used spinal injection, 7- used opioid analgesic	10 (59%) do not need a spinal injection after treatment, 3 decreased the use of opioid analgesics, and 2 completely left using opioid analgesics	N/A	0.30 ± 0.16	0.84 ± 0.12 (180% improvement in general health rating)	3-lead migrations. 1-complained of pain at the IPG
8.	Stidd et al. / 2014 [21]	USA	SCS	9	54.2 ± 4.8	19.9±3.2	VAS 7.2 ± 0.8	2.3 ± 0.9	N/A	N/A	N/A	N/A	66.4% ± 9.3%	N/A	N/A	N/A
9.	Decker et al. / 2017 [26]	UK, Australia, Belgium, USA	Electrical neurostimulation (ReActiv8, Mainstay Medical Limited, Dublin, Ireland)	53	44±10	12	Average back pain NRS: 6.8 ±0.8	NRS:2.4 ±0.4 (57% of subjects had ≥2 point reduction in the single day NRS)	44.9 ± 10.1	14.3 ± 2.3 (60% had ≥10 point improvement in ODI)	N/A	N/A	N/A	0.434 ± 0.185	0.219 ± 0.028 (81% of subjects with ≥0.03 point) after one year	At one year post-activation, there were 145 AEs with 69 (48%) unrelated; and 76 (52%) procedure, device, and/or therapy-related events.
10.	Kapural et al. / 2015 [27]	USA	10-kHz	101 in SCS therapy with 10 kHz, 97- traditional SCS	54.9	12	VAS 7.4 ± 1.2 (10 kHz )and 7.8 ± 1.2 (traditional SCS therapy)	2.5 ± 0.9 (10 kHz) 4.3 (traditional SCS therapy)	HF 10 therapy-ODI: 79.4% of patients Traditional SCS-58.7% of patients	HF10 therapy: ODI: 62.9% of patients Traditional SCS subjects 45.7% of patients	Use of Morphine: 112.7 ± 91.0 mg/day (using 10 khz 125.3 ± 150.0 (using traditional therapy))	87.9 ± 85.2 mg/day (in 10 khZ therapy) 118.0 ± 113.2 mg/day (traditional therapy)	55.4% very satisfied,(10 kHz therapy) 32.4% very satisfied	N/A	N/A	Only 4.0% of HF10 therapy subjects had a study-related serious AE (SAE)

Clinical efficacy and cost-effectiveness

Chronic low back pain has been the major leading cause of disability worldwide and has impaired the QoL for the past several years. Therefore, there is a huge necessity for improving treatment options for those living with that problem and improving their quality of life [27]. SCS therapy with 10 kHz has promising therapeutic action in reducing chronic back and leg pain compared to the traditional method (SENZA-RCT) [20,27]. The 10KHZ HF-SCS (high-frequency-SCS) in the low back pain study showed >50% pain reduction from baseline measured using a 0-100 mm VAS, and 88% of all subjects were not using any opioid compared with 10% at baseline. In addition, 85% of subjects were satisfied, and 50% were in the minimal disability category at 36 months of follow-up [17]. Also, patients' quality-adjusted life-year (QALY) improved by 27% in those who received SCS as compared to those who received conventional medical management (CMM; 12%).

Great patient satisfaction was reported in 88% and even 12% return to their work who undergone SCS treatment, and subjects treated with 10 kHz SCS therapy has improved disability outcomes [28-29]. The cost-utility analysis measured at the starting cost of SCS is higher than conventional medical management (CMM) due to implantation. Once the implantation is done, cost-utility significantly decreases in SCS [29]. The RCT was a study conducted based on cost-utility analysis follow-up; after 3.1 years, the mean cost of success per patient crossed to SCS was $117,901 [30]. Despite no success achieved for the patients who crossed to reoperation, the mean expenditure was $260,584 [30]. A comparative crossover study in three phases of treatment (intention to treat, treated as intended, and final treatment) showed the mean cost per patient of SCS is more effective and less expensive than the patient in reoperation in failed back surgery syndrome (FBSS)-selected patients [30].

Hence, SCS therapy has greater efficacy in controlling chronic pain and improving quality of life. In addition, SCS proves to be cost-effective, as it is less expensive than surgical treatment because once the electrodes are implanted, the cost-utility decreases significantly. Hence, it would be more appropriate in the long term, as the benefit of SCS therapy is much cheaper and effective than other treatment modalities in patients with chronic lower back pain.

Predisposing factors in predicting the success or failure of SCS

Several factors play a role in determining whether spinal cord stimulation will be a success or not in treating low back pain. Identifying these factors could help with appropriate patient selection for this invasive procedure and those patients who can benefit the most from this procedure. In the study by Ondonkor et al., it was analyzed that various factors determine the success or failure of spinal cord stimulation [31]. Success defines as >50% pain relief [31]. The numerous factors are age (p <0.001) - younger patients responded better with a mean age of 54 ± 13 years vs. 64 ± 14 years, primary pain site (p = 0.032) - it was significant in patients with lower extremity pain (86%), stimulator waveform (p = 0.003) - paresthesia-based tonic waveform had the best outcome (46.4%) followed by burst (33.8%), and then paresthesia-free high-frequency waveform (11.3%) [31]. Other variables thought to have an effect were not significant such as diabetes, obesity, opioid use, alcohol, smoking, duration of pain, pain characteristics, and adjuvant medications like anticonvulsants, tricyclics, antidepressants, and number of SCS leads [31].

A retrospective cohort by Cruz et al. shows a significant correlation between smoking (p = 0.017) and recreational/illegal drug use (p = 0.045) with early failure of SCS [32]. The patients who smoked experienced failure due to lead migration or the development of new pain symptoms. It was also found that smokers had more occurrence of depression [32]. The patients who used drugs had to undergo device removal. It was found that body mass index (BMI), depression, and worker's compensation status didn't impact the outcome [32]. It was also seen that there is a significant correlation between the VAS score at one month and six months in the excellent and good groups after SCS surgery is done [33]. The VAS score of the excellent group remained low throughout, whereas the VAS score in the good group increased from one month to six months follow-up (p < 0.05) [33]. However, the VAS score was not statistically significant preoperatively at the baseline between the excellent and good groups [33]. An original study done by Al Jehani et al. showed that intraoperative stimulation (IOS) at the time of SCS surgery determines the success or failure of SCS in the future [34]. A significant difference was observed when IOS was administered for 30-60 minutes between the excellent and good trial outcomes (p = 0.061) [34]. Patients who received IOS for more than 60 minutes experienced high failure rates of SCS (p = 0.0035) [34].

Based on the above information, it can be concluded that the success and failure of spinal cord stimulation therapy depend upon factors like age and different types of stimulator waveform. The other factors include comorbidities, use of oral analgesics, smoking, characteristics and duration of pain, and the number of leads used for the stimulation.

Indications, contraindications, and selection of a patient for spinal cord stimulation therapy

Spinal cord stimulation therapy can be used to manage chronic low back pain, other neuropathic pain, and ischemic pain when other standard treatment methods have failed and the pain persisted for more than six months [35-36]. According to the British Pain Society guidelines, good indications for SCS therapy are those patients with failed back surgery, neuropathic pain especially related to peripheral nerve lesions, and refractory angina pectoris [37]. The group of patients with intercostal neuralgia after thoracotomy, complex regional pain syndrome, pain related to peripheral vascular disease, and neuropathic pain following trauma fall into intermediate indications for SCS therapy. Usually, patients with pain of non-spinal cord origin, postherpetic neuralgia, pain due to the spinal cord injury, and avulsive brachial plexopathy fall are not indicated for SCS therapy [37].

There are contraindications where SCS therapy can't be used, like in patients with a bleeding disorder, patients on anticoagulation therapy, and systemic or local sepsis [3,38]. Patients with psychiatric disorders like psychotic disorder, major depressive disorder, drug and alcohol abusers, and somatization disorder are also not considered good patient selection for the treatment [35,39]. More than 80% of the patients required counseling before SCS therapy as shown in one study [40]. There are some relative contraindications like immune suppression, the presence of cardiac pacemakers, and the use of implanted defibrillators [38]. Pregnant women and patients with inconsistent and abnormal pain may be added to the list of contraindications [3].

Patients selected should understand the process, expense, and invasive nature of therapy [41]. So, during the patients' selection, an assessment should be done by a multidisciplinary team or consulted and approved by at least two physicians with extensive knowledge and experience in pain medicine [35]. The team for the implant should obtain consent from the patients and should perform complete documentation. They should be informed about the expected outcomes, implantation procedures, follow-up time, and requirements and complications. The primary aim of the treatment should be discussed with the patients undergoing treatment, i.e. reduction in the intensity of pain, improved quality of pain after the intervention, reduction in pain medications, and adverse effects [41].

To properly select patients for SCS therapy, the indications and contraindications should be determined carefully with the help group of treating physicians to reduce the harmful effects on the patients and draw the maximum benefits. Proper selection of patients minimizes the unnecessary invasive intervention and maximizes the chance of good outcomes.

## Conclusions

In the past four decades, SCS therapy has achieved good results in reducing the pain of patients with chronic low back pain and improving the functional status of patients. In addition, this type of treatment has shown great promise in treating patients who are not eligible for surgery. However, the increasing prevalence and economic burden should be addressed appropriately to make it easier for patients to obtain SCS treatment. Furthermore, spinal cord stimulation may significantly affect refractory low back pain treatment and benefit clinically considering its technology and mechanism of action. Thus, this therapy will occupy a special place in future, multidisciplinary neuropathic pain management.
